# Impact of the COVID-19 Pandemic on Postoperative Follow-up After a Total Hip and Knee Joints Replacement

**DOI:** 10.1017/dmp.2020.388

**Published:** 2020-10-21

**Authors:** Anis Abobaker, Ifeoluwa Oluwalana

**Affiliations:** Outpatient Department, Spire Fylde Coast Hospital, Blackpool, UK

**Keywords:** COVID-19, total joints replacement, remote consultation

Patients who have undergone a total hip or knee joints replacement get a face-to-face appointment with their orthopedic surgeons 3 to 6 weeks after the operation (as per hospital protocol) to assess their postoperative progress. The main clinical parameters that are assessed during the consultation are pain control, mobility and degree of joint flexion and extension, signs of deep vein thrombosis (DVT), wound healing, and signs of wound infection, which means that performing a clinical examination is very important to efficiently assess the above clinical parameters. However, since the start of the 2019 coronavirus disease (COVID-19) pandemic, there has been a shift toward using remote consultation instead of the traditional face-to-face consultation to limit the extent of doctor and patient interaction and reduce the risk of transmission of infection.^1^ To assess how effective remote consultation is for postoperative follow-up after a total hip and knee joints replacement during the pandemic compared with a face-to-face consultation, using a questionnaire format, we asked 5 orthopedic consultants working at Spire Fylde Coast Hospital (Blackpool, the United Kingdom) about their experience with postoperative follow-up by remote consultation.

The responses of the orthopedic consultants to the questionnaire are summarized in Table 1. The postoperative follow-up clinics were conducted by telephone consultation. All the 5 consultants declared that face-to-face consultation is more effective. They stated that the main limitation of the telephone consultation is the inability to perform a clinical examination, which makes it hard to assess the clinical progress objectively. In addition, telephone consultation does not have the same extent of human interaction compared with the traditional face-to-face method of consultation. This might indicate that an alternative approach of remote consultation is required, such as performing a video consultation; 3 out of the 5 consultants stated that the outcome would have been better if the consultations were conducted by this technique.

**Table 1.** Responses of the orthopedic consultants to the questionnaire regarding remote consultation for postoperative follow-up following a hip and knee joints replacement

	Consultant 1	Consultant 2	Consultant 3	Consultant 4	Consultant 5
How was the remote consultation conducted?	Telephone consultation	Telephone consultation	Telephone consultation	Telephone consultation	Telephone consultation
Telephone vs video consultation (which one is more effective?)	Video consultation	Video consultation	Video consultation	Telephone consultation	Telephone consultation
Remote consultation vs face-to-face consultation (which one is more effective?)	Face-to-face consultation	Face-to-face consultation	Face-to-face consultation	Face-to-face consultation	Face-to-face consultation
Reasons why remote consultation is less effective than face-to-face consultations?	*Lack of clinical examination makes it difficult to assess signs of DVT and mobility as the differentiation between subjective and objective findings becomes difficult with remote consultation *difficult to assess pain severity as it is difficult to differentiate between localised and referred pain	*Lack of clinical examination makes it difficult to assess wound adequately and to assess signs of DVT *Not the same extent as human interaction	*Lack of clinical examination makes it difficult to assess wound healing, signs of DVT, range of movements of the joints and gait	*Lack of clinical examination makes it difficult to assess signs of DVT or wound infection	*Cannot perform physical examination
Were you satisfied with the outcome of the remote consultation?	No	No	No	Yes	Yes
The easiest clinical parameter to assess via remote consultation	Wound	Mobility	Pain	Mobility	Mobility
The most difficult clinical parameter to assess via remote consultation	Pain	Wound	Wound	Wound	Pain
Were all patients accessible via remote consultation?	No	No	Yes	No	No

In addition to reducing the risk of transmission of infection during the current pandemic, remote consultation for postoperative follow-up has other advantages.^2^ Risk stratification of patients can be performed during the remote consultation. For instance, patients mentioning concerning signs of wound infection or DVT, such as redness around the wound or calf pain, can be booked to come to the clinic for an imminent clinical examination.^1,2^ This approach limits the number of patients coming to the outpatient clinic, which in turn reduces the utilization of health care resources during this difficult time.^3^ Sharareh et al. used Skype video calls, in addition to the scheduled in-clinic appointments, to follow-up patients after total joints arthroplasty during the first 2 months of the postoperative period.^3^ The follow-up Skype calls were made 5 times during the first, third, fourth, sixth, and ninth postoperative weeks, and each clinical consultation was less than 3 minutes. These multiple and short Skype calls significantly reduced the number of unscheduled in-clinic visits and postoperative phone calls.^3^

As the lack of a clinical examination was the main limitation of a telephone consultation, the principle of the virtual orthopedic examination could partially solve this problem.^4^ It is conducted by giving patients pre-clinic instructions about camera positioning and location that enables full exposure and inspection of the joints.^4^ The degree of joint movement can be objectively assessed using web-based goniometer.^4^ This technique can be challenging for elderly patients given the advanced technology required in this procedure, as well as for patients who have no access to the Internet.

As only 2 out of 5 consultants were satisfied with the outcome of telephone consultation because of the inability to perform a clinical examination, it seems that the approach for postoperative follow-up by telephone consultation at our hospital might require improvement. Video consultation by the Zoom application can be a feasible alternative as it provides visual interaction between doctors and patients. In addition, at least the inspection part of the clinical examination can be performed, such as assessment of gait, wound healing, and signs of wound infection. However, the technicality and limited access to the Internet are the main challenges for this type of remote consultation.

In summary, it seems that the COVID-19 pandemic has a significant impact on the quality of the postoperative follow-up after a total joint replacement. The main limitation of our study is the small number of consultants who responded to the questionnaire. Further studies on a larger scale are required to assess the external validity of our findings.
